# Lung aeration on post-mortem magnetic resonance imaging is a useful marker of live birth versus stillbirth

**DOI:** 10.1007/s00414-014-1125-7

**Published:** 2014-12-05

**Authors:** Joy L. Barber, Neil J. Sebire, Lyn S. Chitty, Andrew M. Taylor, Owen J. Arthurs

**Affiliations:** 1 Department of Radiology, Great Ormond Street Hospital for Children NHS Foundation Trust, London, UK WC1N 3JH; 2 Department of Histopathology, Great Ormond Street Hospital for Children NHS Foundation Trust, London, UK; 3 UCL Institute of Child Health, London, UK; 4 Genetics and Genomic Medicine, UCL Institute of Child Health, London, UK; 5 Great Ormond Street Hospital for Children NHS Foundation Trust, London, UK; 6 UCLH NHS Foundation Trust, London, UK; 7 Cardiorespiratory Division, Great Ormond Street Hospital for Children NHS Foundation Trust, London, UK; 8 Centre for Cardiovascular Imaging, UCL Institute of Cardiovascular Science, London, UK

**Keywords:** Post-mortem, Foetal, MR, Lung aeration

## Abstract

**Objective:**

Aim of this study was to investigate whether lung assessment on post-mortem magnetic resonance imaging (PMMR) can reliably differentiate between live birth and stillbirth.

**Materials and methods:**

We retrospectively assessed PMMR imaging features of a group of late foetal terminations following fetocide and stillbirths (without witnessed breathing) and early infant deaths (breathed spontaneously before death). PMMR images were reviewed for evidence of lung aeration and other features, blinded to the clinical and autopsy data.

**Results:**

Nineteen infant deaths (mean age 3.0 ± 6.5 post-natal weeks) and 23 foetal terminations or stillbirths (mean age 32.6 ± 10.2-week gestation) were compared. Subjective appearances of lung aeration on PMMR were the best indicator of live birth, with a sensitivity of 89.5 % (95 % confidence interval 68.6, 97.1 %) and specificity of 95.6 % (79.0, 99.2 %) and positive and negative predictive values of 94.4 % and 91.7 %, respectively.

**Conclusions:**

Lung aeration on PMMR appears to have high overall accuracy for confirmation of live birth versus intrauterine foetal death but now requires validating in a larger cohort of perinatal deaths.

## Introduction

Establishing whether an infant was stillborn, or was born alive and subsequently died, is a difficult but important aspect of forensic medicine. Traditional methods for assessing lung aeration include the lung flotation technique, an invasive test requiring evisceration of the lungs and observing whether they float or sink when placed into water; floating lungs are traditionally deemed to contain air, indicating breathing before neonatal demise [1]. However, there is uncertainty regarding the reliability of this test, with various factors potentially contributing to both false positive and false negative findings [2, 3]. There is a paucity of published studies on the accuracy of the lung flotation test, with quoted accuracy ranging from 37 to 95 % [3, 4].

Post-mortem imaging is becoming widely accepted as an important component of the examination after death [5, 6], with excellent diagnostic agreement between perinatal and paediatric post-mortem magnetic resonance imaging (PMMR) and autopsy findings [7]. Part of the challenge of post-mortem (PM) imaging is the correct interpretation of imaging findings, and two small case series (11 cases in total) have described the use of post-mortem computed tomography (PMCT) to assess lung aeration in infant deaths [8, 9]. However, PMMR provides much better soft tissue detail than PMCT in foetuses, stillbirths and neonates and is likely to become the preferred imaging technique in this patient population [7, 10].

In this study, we investigate the accuracy of PMMR imaging characteristics to distinguish between liveborn and stillborn infants to establish a non-invasive alternative or adjunct to the lung flotation test, which could be more acceptable to parents who decline an invasive autopsy, and to more accurately interpret routine PMMR imaging.

## Methods

We retrospectively reviewed our hospital database for cases of late foetal death, termination of pregnancy, and infant deaths in the first 3 months of life, who had undergone PMMR as part of their post-mortem assessment in the last 5 years. At our large specialist paediatric hospital, we perform over 100 foetal and paediatric PMMR studies per year as part of the clinical autopsy. This study did not require further specific institutional approval, as all parents had consented to a clinical pre-autopsy PMMR as part of our institution’s clinical post-mortem assessment, and use of routinely collected post-mortem data is approved by the local research ethics committee. Bodies were stored in a mortuary at 4 °C, and PMMR was generally performed out of hours, causing least disturbance to clinical services.

Our inclusion criteria identified two groups: group 1: “live births” defined as infants who were born alive and witnessed spontaneously breathing before death, and group 2: “non-live births” defined as either late foetal terminations of pregnancy for abnormality following fetocide (over 24-week gestation) without documented evidence of breathing and stillbirths who had been born dead. We excluded all foetuses below 24-week gestation, as they were likely to have incomplete lung development, and also excluded cases with lung abnormalities at autopsy.

### PMMR imaging

Pre-autopsy whole-body PMMR was acquired using a 1.5T MR scanner (Avanto, Siemens Medical Solutions, Erlangen, Germany). Sequences acquired included whole-body 3-D T_2_-weighted turbo spin echo (TSE, TR 3500 ms, TE 276 ms, voxel size 0.8 × 0.8 × 0.8 mm, two averages), 3-D T_1_-weighted volumetric interpolated breath-hold examination (VIBE; TR 5.9 ms, TE 2.4 ms, flip angle 25°, voxel size 0.8 × 0.8 × 0.8 mm, eight averages), and 3-D constructive interference in the steady state (CISS) sequence (TR 9.2 ms, TE 4.6 ms, flip angle 70°, voxel size 0.6 × 0.6 × 0.6 mm, four averages). The VIBE sequence was chosen, as it was particularly sensitive to the presence of gas due to the associated chemical shift artefact. No exogenous contrast agents were applied.

PMMR findings were reviewed by two radiologists with 3- (JB) and 7-year (OA) clinical radiology experience (0- and 2-year PMMR reporting experience, respectively), using the OsiriX platform (OsiriX Foundation, Geneva, Switzerland), blinded to the clinical history and autopsy findings. A consensus decision was reached regarding the presence of any air (very low signal intensity) identified on both T_1_-weighted and T_2_-weighted sequences in the following six anatomical locations: in (1) airways, (2) lung parenchyma, (3) gastrointestinal tract, (4) right heart cavities, (5) left heart cavities, and (6) hepatobiliary system. The presence of post-mortem pleural fluid collections was also recorded but predicted to be consistent across the two groups (a control measure), as this was assumed to be unrelated to breathing.

### Autopsy

Conventional autopsies were performed by one of several experienced specialized perinatal/paediatric pathologists in accordance with national guidelines and were reported blinded to the PMMR findings. Autopsy findings were reviewed for this study to exclude any cases which had abnormal lung pathology. We used the contemporaneous clinical history and perinatal notes as the gold standard for assessing breathing status.

### Data and statistical analysis

Primary outcomes were sensitivity, specificity, positive predictive value (PPV) and negative predictive value (NPV) where PMMR (the index test) was compared with clinical history (the gold standard), with 95 % confidence intervals (CIs). Concordance was defined as the sum of true positives and true negatives divided by all cases. Exact methods were used to calculate CIs [11]. SPSS (Version 19 for Macintosh, SPSS Inc., IBM, New York, USA) was used for data analysis.

## Results

### Demographics

We found 42 cases in our database which met our strict inclusion criteria from our hospital database of over 500 cases (2007–2012).

Group 1 live births comprised 19 infant deaths (mean post-natal age at death 3.0 ± 6.6 weeks). Sixteen breathed spontaneously at birth and had lived for up to 11 weeks prior to death, which was attributed to a variety of causes including trauma and sudden unexpected death in infancy (SUDI). Three further cases had very low APGAR scores at birth but underwent resuscitation with a short period of supported life prior to death at 24–48 h of age.

Group 2 non-live births comprised 23 foetal terminations or stillbirths (mean age 32.6 ± 10.2-week gestation) of which 17 were late foetal terminations (known to have not breathed and not undergone resusCitation) and six stillbirths without spontaneous breathing.

### Imaging

Gas in almost all locations, including airways, lungs, or GI tract, was identified more commonly in live births than stillbirths (Table 1).

**Table 1:** Diagnostic accuracy of imaging indices for live birth

Index	FP/TP	FN/TN	Sensitivity (%)	Specificity (%)	PPV (%)	NPV (%)	Concordance (%)
Gas in airway	7/16	3/16	84.2 % (62.4, 94.5)*	69.6 % (49.1, 84.4)	69.6 % (49.1, 84.4)	84.2 % (62.4, 94.5)	76.2 % (61.5, 86.5)
Lung aeration	1/17	2/22	89.5 % (68.6, 97.1)	95.6 % (79.0, 99.2)	94.4 % (74.2, 99.0)	91.7 % (74.2, 97.7)	92.9 % (81.0, 97.5)
Gas in GI Tract	5/19	0/18	100 % (83.2, 100)	78.3 % (58.1, 90.3)	79.2 % (59.5, 90.8)	100 % (82.4, 100)	88.1 % (75.0, 94.8)
Absence of R Ht gas	9/14	5/14	73.7 % (51.2, 88.2)	60.9 % (40.8, 77.8)	60.9 % (40.8, 77.8)	73.7 % (51.2, 88.2)	66.7 % (51.6, 79.0)
Absence of L Ht gas	8/17	2/15	89.5 % (68.6, 97.1)	65.2 % (44.9, 81.2)	68.0 % (48.4, 82.8)	88.2 % (65.7, 96.7)	76.2 % (61.5, 86.5)
Absence of hepatobiliary gas	12/16	3/11	84.2 % (62.4, 94.5)	47.8 % (29.2, 67.0)	57.1 % (39.1, 73.5)	78.6 % (52.4, 92.4)	64.3 % (49.2, 77.0)
Bilateral effusions	11/10	9/12	52.6 % (31.7, 72.7)	52.2 % (33.0, 70.8)	47.6 % (28.3, 67.6)	57.1 % (36.6, 75.5)	52.4 % (37.7, 66.6)

A positive test result was defined as the presence of any aeration in the given location

*FP* false positive, *TP* true positive, *FN* false negative, *TN* true negative, *R Ht* right heart (atrium or ventricle), *L Ht* left heart (atrium or ventricle)

*All percentages are given ±95 % confidence interval

**Fig. 1:**
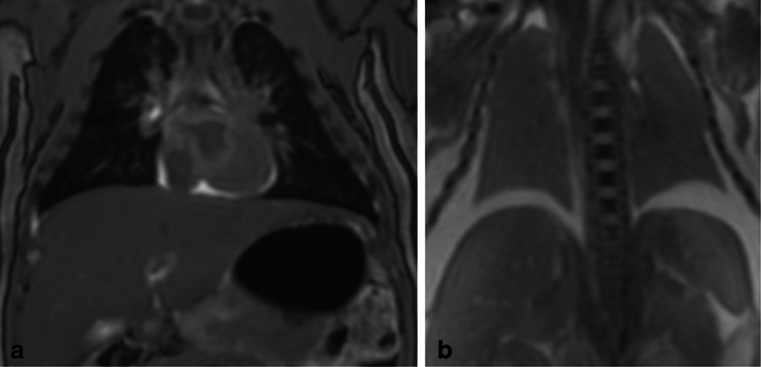
Example of agreement between PMMR and autopsy. **a** Coronal T_2_-weighted PMMR image in a 2-week-old neonate who fell from the pram and died. It demonstrates air within the airways and lungs (*dark*) and gas within the stomach (*dark*), as would be expected in an infant who had breathed before death (true positive in this study). **b** Coronal T_2_-weighted PMMR image in a 30-week-old gestation foetus who underwent termination for foetal hydrops. There were no signs of life at delivery, no resuscitation implemented, and PMMR demonstrated no gas in the lungs (*grey* lungs), nor GI tract (not shown), as would be expected (true negative in this study)

Lung aeration on PMMR was the best indicator of live birth, with highest concordance between PMMR and autopsy of 92.9 % (95 % CI 81.0, 97.5). Lung aeration was the most specific marker of live birth, with specificity of 95.6 % (79.0, 99.2) and PPV of 94.4 % (74.2, 99.0), whereas gas in the GI tract was the most sensitive at 100 % (86.2, 100) and highest NPV 100 % (82.4, 100) (Fig. 1).

Intracardiac gas and hepatobiliary gas were more commonly seen in stillbirths than in live births, but, together with the presence of pleural effusions, were not reliable indicators of live birth versus stillbirth status.

**Fig. 2:**
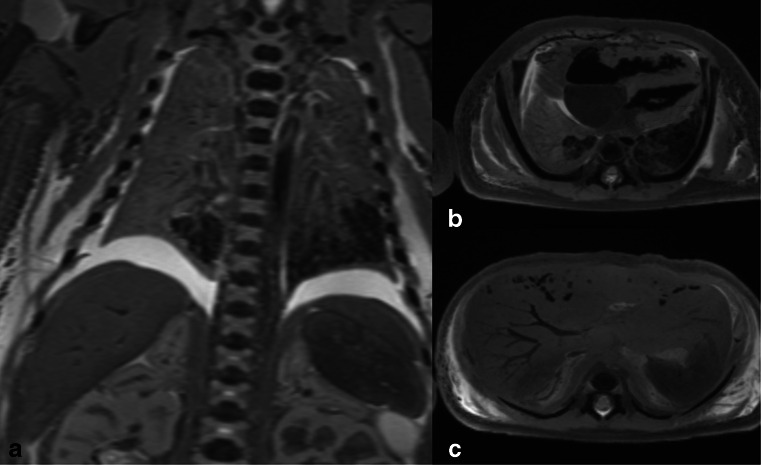
Example of false positive PMMR. PMMR of an unexplained stillbirth at 38-week gestation. A faint foetal heart rate was thought to be detected prior to emergency caesarean section, but there was evidence of intrauterine death and maceration at delivery. Extensive resuscitation was given at birth including intubation, cardiopulmonary resuscitation and adrenaline. T_2_-weighted PMMR imaging shows that the lung bases contain air (*dark*) on both coronal (**a**) and axial images (**b**) and gas within the hepatobiliary system (**c**). We suggest that these findings may be related to initial resuscitation rather than spontaneous breathing

For lung aeration, 17 true positive and 22 true negative diagnoses were made on PMMR. A single false positive diagnosis was made where apparently well-aerated lungs were seen in a stillborn infant (Fig. 2). The clinical history recorded no witnessed spontaneous breathing at 38-week gestation; however, this foetus may have undergone resuscitation attempts and the air within the lungs may be related to mechanical ventilation.

**Fig. 3:**
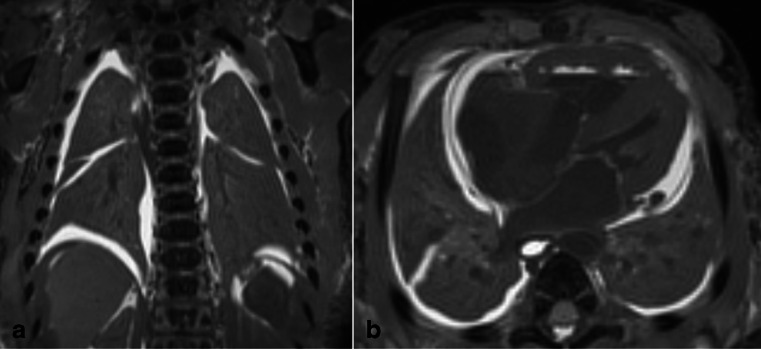
Example of false negative PMMR. Coronal (**a**) and axial (**b**) T_2_-weighted PMMR imaging of the lungs in a 2-day-old neonate who died of ischaemic brain injury. The baby had no heart rate at birth and very poor APGAR score and was resuscitated and mechanically ventilated but care was withdrawn. Despite the ventilation, the lungs did not contain air on PMMR. Interestingly, we also did not see gas in the airway (not shown)

Two false negative diagnoses on PMMR were made, where neonates had documented spontaneous breaths, but no gas was seen in the lung parenchyma (Fig. 3). Both were neonates who died from perinatal asphyxia and ischaemic brain injury in the first week of life.

As it was possible that the use of “both lung and GI tract aeration” or “either lung or GI tract aeration” together could improve the diagnostic accuracy of PMMR, we repeated the statistical analysis for both of these measures. Both of these analyses gave the same results as those for lung aeration only and did not improve the diagnostic accuracy.

## Discussion

Our results indicate that subjective lung parenchyma aeration on PMMR is a good indicator of spontaneous breathing and can be useful to distinguish live birth from stillbirth in most cases. This method of assessing stillbirth has similar accuracy to traditional lung flotation methods but has the primary advantage of being completely non-invasive and may be acquired along with routine PMMR imaging. With the increasing use, experience and acceptability of non-invasive imaging-based post-mortem examinations in addition to conventional invasive autopsy techniques, PMMR can give reliable information about the body without the need for invasive testing.

Our results are similar to the most recent formal study of the lung flotation test by Groβe Ostendorf et al. [4] which included 208 cases, in which they report 98.0 % (95.1–99.3 %) concordance, with sensitivity of 71.4 % (45.4–88.3 %), specificity of 100 % (98.0–100 %), PPV 100 % (72.3–100 %) and NPV 98.0 % (94.9–99.2). They had no false positives (lungs of a stillborn which float) and four false negatives (lungs of a live birth which sink). They argue that “a negative test result cannot be taken as proof for a newborn never to have breathed at all”. In keeping with their results, we found an absence of air in the lungs in two live births (two false negatives), with specificity of lung aeration for live birth higher than sensitivity. However, 194 of their 208 cases were born dead, and 125 of these were below 22-week gestation—which we excluded in our study due to the potential for confounding due to lung immaturity and the relative lack of clinical relevance in this population. The very large proportion of stillbirths and low gestation of some of their cases may have contributed to their higher specificity.

In all studies of this type, uncertainty will remain over the factors such as the accurate identification of successful spontaneous breathing and, in particular, over the role of resuscitation in introducing air into the lungs of stillbirths. In this study, we attempted to control as many variables as possible, with most cases either terminations who had definitely not breathed and not been resuscitated and early infant deaths who had unequivocally breathed spontaneously. In real clinical scenarios, the situation is rarely so controlled, and it remains to be seen how much aeration that a few gasps or brief attempts at initial resuscitation could introduce into the lungs. The main clinical circumstance in which this information is important is to distinguish stillbirth from live birth in cases of suspected infanticide [12] and medico-legal cases in which there is uncertainty regarding optimal management of labour and delivery. Resuscitation as a confounder in this context could be tested by attempting to ventilate known stillborn infants in future studies. In adults, post-mortem imaging has been performed during lung ventilation (ventilated PMCT) in order to differentiate collapsed post-mortem lungs from true pathology [13], and the introduction of this technique into perinatal PM imaging may allow resolution of this issue.

We primarily identified the presence or absence of gas on whole-body 3-D T_2_-weighted turbo spin echo (TSE) PMMR sequences, which can give excellent signal to noise at high resolution in these cases. Air is typically black or very low signal intensity compared to the surrounding tissues and is usually easy to identify in particular within the lungs. Where there was doubt, other sequences can be useful; for instance, we found the VIBE sequence useful, as it is particularly sensitive to the presence of gas due to its chemical shift artefact. We consider a 3-D T_2_-weighted and 3-D T_2_-weighted sequence of the body to be an appropriate minimum acquisition standard in foetal and paediatric PMMR body imaging, as they should allow clinical abnormalities to be identified with confidence, and reconstruction in any plane.

In a medico-legal context, both false positives and false negatives can have equally significant consequences. A false positive result (interpreting a stillbirth as having breathed) could incorrectly lead to accusations of an innocent caregiver of infanticide/neonaticide, whereas a false negative result (incorrectly interpreting a live birth as having no lung aeration) could inaccurately provide a defence to a guilty party. In this context, as consent from the parents is typically not required when they fall under the jurisdiction of those investigating a suspicious death, formal autopsy tests are likely to remain the gold standard. However, with the recent decline in parental acceptance of invasive autopsy, there has been increasing emphasis on the accurate interpretation of perinatal and paediatric PM imaging as part of a more minimally invasive approach. Part of the challenge of PM imaging is the correct interpretation of imaging findings, which this study will help to educate and elucidate further.

Despite this being the only study to have addressed this issue using PMMR, and our study population being larger than other studies using PM CT [8, 9], sample size remains a limitation. We were unable to further evaluate the role of resuscitation in potentially generating false positive PMMR results. Furthermore, our data is based on the accuracy of clinical data regarding witnessed spontaneous breathing or definite stillbirth (although for the vast majority of cases, this was clearly documented). A further limitation is that we also excluded those with congenital lung abnormalities or lung disease such as pneumonia, in which the presence of additional pathology could affect these results. The diagnosis of pneumonia on PMMR is particularly challenging [14], and future studies will be needed to clarify how various pathological states (pneumonia, haemorrhage etc.) and physiological changes (post-mortem fluid accumulation) could affect these imaging investigations.

## Conclusion

In summary, these findings demonstrate a high overall accuracy for determination of lung aeration by PMMR as an indicator for spontaneous breathing in foetal and early infant deaths. This approach has a similar accuracy to traditional invasive tests, but is non-invasive, and therefore likely to become more acceptable in future autopsies which involve PMMR as a standard investigation.
